# Unveiling a Novel Source of Resistance to Bacterial Blight in Medicinal Wild Rice, *Oryza officinalis*

**DOI:** 10.3390/life12060827

**Published:** 2022-06-02

**Authors:** Ling Chen, Fuyou Yin, Dunyu Zhang, Suqin Xiao, Qiaofang Zhong, Bo Wang, Xue Ke, Zhiyuan Ji, Lingxian Wang, Yun Zhang, Cong Jiang, Li Liu, Jinlu Li, Yuanda Lu, Tengqiong Yu, Zaiquan Cheng

**Affiliations:** 1 Biotechnology and Germplasm Resources Institute, Yunnan Academy of Agricultural Sciences, Yunnan Provincial Key Lab of Agricultural Biotechnology, Ministry of Agriculture, Kunming 650205, China; cl@yaas.cn (L.C.); yinfuyou2007@126.com (F.Y.); zhangdunyu@163.com (D.Z.); xiaosuqin227@126.com (S.X.); zqf820101@aliyun.com (Q.Z.); wangbo1040@163.com (B.W.); ke-xue@hotmail.com (X.K.); wanglingxian2001@aliyun.com (L.W.); zhangyun507@163.com (Y.Z.); fengjinny@163.com (C.J.); liuliyaas@163.com (L.L.); li_jinlu@yeah.net (J.L.); yuanda@163.com (Y.L.); 2 National Key Facility for Crop Gene Resources and Genetic Improvement (NFCRI), Institute of Crop Sciences, Chinese Academy of Agriculture Sciences (CAAS), Beijing 100081, China; jizhiyuan@caas.cn; 3 College of Plant Protection, Yunnan Agricultural University, Kunming 650201, China

**Keywords:** bacterial blight, *Oryza officinalis*, resistance, wild rice, medicinal rice

## Abstract

Bacterial blight (BB) caused by *Xanthomonas oryzae* pv. *oryzae* (*Xoo*) is among the oldest known bacterial diseases found for rice in Asia. It is the most serious bacterial disease in many rice growing regions of the world. A total of 47 resistance (R) genes (*Xa1* to *Xa47*) have been identified. Nonetheless, these R genes could possibly be defeated to lose their qualitative nature and express intermediate phenotypes. The identification of sources of novel genetic loci regulating host plant resistance is crucial to develop an efficient control strategy. Wild ancestors of cultivated rice are a natural genetic resource contain a large number of excellent genes. Medicinal wild rice (*Oryza officinalis*) belongs to the CC genome and is a well-known wild rice in south China. In this study, *O. officinalis* was crossed with cultivated rice HY-8 and their hybrids were screened for BB resistance genes deployed through natural selection in wild rice germplasm. The molecular markers linked to R genes for BB were used to screen the genomic regions in wild parents and their recombinants. The gene coding and promoter regions of major R genes were inconsistently found in *O. officinalis* and its progenies. *Oryza officinalis* showed resistance to all thirty inoculated *Xoo* strains with non-availability of various known R genes. The results indicated the presence of novel genomic regions for BB resistance in *O. officinalis*. The present study not only provides a reference to investigate medicinal rice for R gene(s) identification against BB but also identified it as a new breeding material for BB resistance.

## 1. Introduction

Bacterial blight (BB) caused by *Xanthomonas oryzae* pv. *oryzae* (*Xoo*) is amongst the oldest known bacterial diseases in Asia [1,2]. It is the most serious bacterial disease in many rice growing regions of the world [3]. The *Xoo* strain enters through hydathodes, stomata and wounds on the roots or leaves which causes leaf wilting, affects photosynthesis that results in yield loss and can reduce rice yield by as much as 20–80% [4]. BB causes serious loss of rice production in Asia, Australia, Latin America, Africa and the United States [5,6,7]. It is particularly destructive in the rice growing tracts of Asia during monsoon season. At the seedling stage under high atmospheric temperatures (28–34 °C) sometimes *Xoo* infection causes the death of the central shoot, leading to complete crop loss [1,8].

*Xoo* isolates collected from and across Asia, Africa, and Australia exhibit high genetic diversity based on the polymorphism of transposable elements, a-virulence genes, insertion sequences, rep/box elements and other markers [1,9]. Based on the virulence of *Xoo* strains in particular host genotypes, several distinct races have been identified [1,10]. Around 30 races of *Xoo* have been reported globally [1,11]. Studies on *Xoo* pathotype diversity revealed 6–11 pathogenic races based on their virulence to *Xa/xa* differential lines only in India [1,11,12]. Among the total known resistance genes, *Xa4*, *xa5*, *Xa7*, *xa8*, *Xa11*, *xa13* and *Xa21* should be targeted as important candidates for resistance breeding against BB races in southwestern Asia.

There are different conventional control measures of BB such as antibiotics and application of copper compounds. The increasing trend of rice monoculture has spurred the development and emergence of new and more virulent races of *Xoo*, causing ineffectiveness of most of the chemical means of disease control. However, the development of resistant cultivars by incorporating major resistance (R) gene(s) has been proved to be the most effective, economical and eco-friendly strategy to control BB.

A total of 47 R genes (*Xa1* to *Xa47*) have been identified [1,13]. Out of these, 14 are recessive genes, while some display semi-dominance (e.g., *Xa27*). Fourteen of the total R genes such as *Xa1*, *Xa3/Xa26*, *xa5*, *xa13*, *Xa10*, *Xa21*, *Xa23*, *xa25*, and *Xa27* have been cloned and characterized indicating the involvement of multiple mechanisms of R-gene-mediated *Xoo* resistance [2,14]. The majority of the R genes have been tagged with closely linked molecular markers and are being used in marker-assisted selection for gene pyramiding [1,2,14]. Some genes, e.g., *Xa21*, *Xa22*, *Xa23*, *Xa3/Xa26*, *Xa31*(*t*) and *Xa39* confer resistance to a broad spectrum of *Xoo* races, whereas others are effective against a limited number of localized BB races. *Xoo* race-specific resistance in rice is controlled by both major R genes with qualitative effect, and by quantitative trait loci (QTL) that condition for partial resistance [1,15]. The R genes could possibly be defeated to lose their qualitative nature and express intermediate phenotypes [1,16].

The identification of sources of novel genetic loci regulating host plant resistance is crucial to develop an efficient strategy followed by screening, mapping, cloning and breeding. The search for a novel source of resistance is a continuous process, as the breakdown of resistance occurs due to the appearance of virulent *Xoo* races [5,17]. With ever-evolving pathogens and changing climate patterns, it is now essential to know the status of the resistance gene(s), to expand genetic resources with novel BB resistance genes, and to deploy and pyramid them in breeding programs for durable resistance to *Xoo*. Identification and isolation of novel host resistance and pathogen a-virulence genes are required for a broader understanding of mechanisms involved in host–pathogen interactions and also to determine the resistance breeding approaches. The wild species often contain untapped resources of distinct alleles useful for breeding programs. For this purpose, normally tightly linked molecular markers are exploited in order to identify genotypes with multiple resistance genes. Molecular markers offer an opportunity to characterize the germplasm collections for the existence of various resistance genes. Several markers specific to BB resistance genes have been previously studied [5,17,18,19,20]. The marker aided selection (MAS) approach has proved its efficiency in breeding programs to improve rice genotypes against disease which allows the introgression/pyramiding of single/multiple resistance genes in a genotype with desirable traits [5,6,17,21].

Wild ancestors of cultivated rice, a natural genetic resource contain a large number of excellent genes. Along with ordinary wild rice (*Oryza rufipogon* Griff.), the granular wild rice (*O. meyeriana* Baill.) and the pharmaceutical/medicinal wild rice (*O. officinalis* Wall.) are also well-known genetic resources. Different species are categorized into 10 genome types, six are diploid (AA, BB, CC, EE, FF, and GG) (2n = 2x = 24) and the other four are allotetraploid (BBCC, CCDD, HHJJ, and HHKK) (2n = 4x = 28) [22]. Medicinal wild rice (*O. officinalis*) belongs to the CC genome. The *O. officinalis* genome is 1.6 times larger than the AA genome of cultivated *O. sativa*, mostly due to proliferation of Gypsy type long-terminal repeat transposable elements, but overall syntenic relationships are maintained with other *Oryza* genomes (A, B, and F) [17]. With its diverse ecology, it has been distributed in the Yunnan, Guangdong, Guangxi and Hainan provinces of China. Its higher genetic diversity has resulted in higher resistance and tolerance to many diseases including BB.

In this study, the *O. officinalis* genotypes from the Yunnan province of China were crossed with *O. sativa* subsp. *indica* HY-8 and their hybrids were screened for BB resistance genes deployed through natural selection and molecular selection in wild rice germplasm. The wild rice demonstrated BB resistance in the absence of major known BB-resistance genes. The exploration of BB in its descendants provides a theoretical basis and data support. This information will aid in the further utilization of the wild rice germplasm, and in deciding gene pyramiding programs for BB resistance genes in high yielding rice varieties.

## 2. Material and Methods

### 2.1. Plant Materials

**Figure 1:**
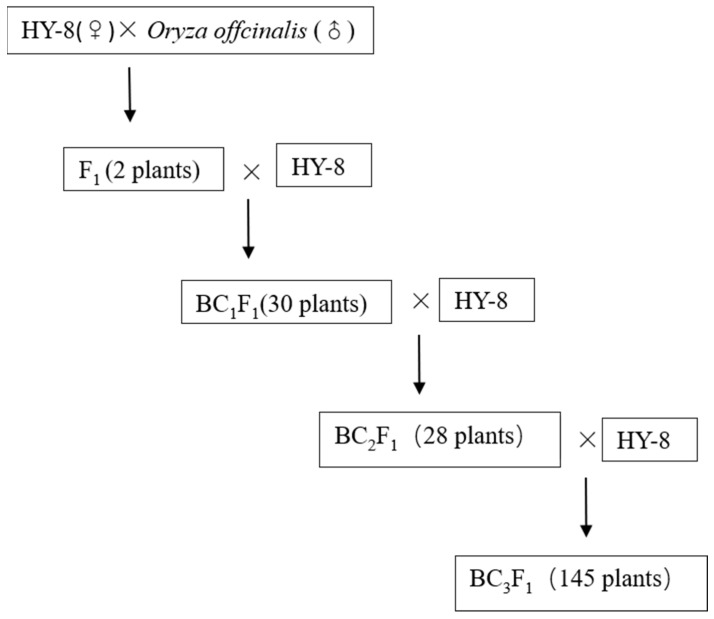
Establishment of mapping population for bacterial blight from medicinal wild rice *Oryza officinalis* and the domesticated rice HY-8.

The *O. officinalis* from Mingding, Yunnan Province of China were obtained to hybridize with *O. sativa* subsp. *indica* HY-8. The F_1_ was further crossed and selfed to obtain the next segregation generations. A total of 30 BC_1_F_1_ individuals were obtained for both of the F_1_ hybrids and HY-8 progeny, respectively. The 28 BC_2_F_1_ and 145 BC_3_F_1_ plants, 4 F_2_ generation individuals were finally obtained from the crossing of F_1_ for further study. A local japonica rice cultivated variety ‘02428’ was used as a susceptible control denoted as “Control” during our evaluation (Figure 1, Supplementary Table S1).

### 2.2. Pathogen Collection for Inoculation

A total of 30 domestic and international pathogenic bacterial strains were collected for this study (Supplementary Table S1). Among them, C1 to C7 and C9 were obtained from local research institutes; T7147 and PXO99^A^ were the international strains; PB, was a PXO99^A^ mutant strain; Y8, X1, X6, X9 and X10, were local pathogenic bacteria of Yunnan; HZ, Hzhj19, YM1, YM187, YJdp-2 and YJws-2, were obtained from fresh leaf samples from different rice areas of Yunnan in epidemic conditions; LN44, HAN05-1, HAN08-2, YuN17-1, YuN18-2, YuN96-11 and YuN98-5 were obtained from the Institute of Crop Science, Chinese Academy of Agricultural Sciences, Beijing, China.

The inocula of all 30 strains were stored at −80 °C. Before use, the pathogens were cultured on Na-solid medium, at 28 °C for 48 h to 72 h, eluted with sterile water, formulated into a concentration of 3 × 10^8^ CFU/mL with 0.5 OD of 600 visible wavelengths.

### 2.3. Pathogen Inoculation and Screening of Germplasm

The selected germplasm was grown in a field at Yumen, Yunnan Province, China screening base during the wet season (May to July) of 2019 and 2020. Each genotype was grown in a single row plot with a standard plant-to-plant and row-to-row distance of 10 cm and 15 cm, respectively. The plants were inoculated with the propagules of a BB field isolate using the leaf-clipping inoculation method [1,23]. During the reproductive growth stage, five fully expanded uppermost leaves from five plants of each entry were clip inoculated at around six weeks after transplanting (i.e., at the maximum tillering stage). The pathogen growth on the local *japonica* variety susceptible to BB was considered as a control. The lesion lengths (LL) were recorded after about three weeks on a single leaf from each of the five inoculated plants. BB severity (growth of the lesions) was visually scored following the Standard Evaluation System (SES) for rice [1,24]. That is, a lesion length less than or equal to 6 cm is resistant (R), and a lesion length greater than 6 cm is susceptible (S). Each of the calculated data points was the average of 15 measured readings.

### 2.4. Identification of R Genes

A total of 63 primer pairs were obtained to evaluate the 15 BB resistance genes. They are used for marker assisted selection in wild rice germplasm. The genomic sequences of 13 BB resistance genes (*Xa1*, *Xa2*, *Xa3*, *Xa4*, *Xa5*, *Xa7*, *Xa10*, *Xa13*, *Xa14*, *Xa21*, *Xa23*, *Xa25* and *Xa26*) were obtained from an online rice database to design the specific primers, while *Xa32*(*t*), *xa34*(*t*), *Xa38*(*t*), *xa42*(*t*) and *Xa45*(*t*)_11_ functional markers were used as reported (Table 1). Three to nine primer pairs were designed to cover the whole length of each gene. Total genomic DNA was extracted from fresh leaves as per the standard protocol provided by the DNA-extraction kit (Tiangen Biochemical Technology, Co., Ltd., Beijing, China). The high-quality DNA was separated in 1% agarose gel and the DNA concentration was controlled by A260/A280 spectroscopy values.

The polymerase chain reaction (PCR) was performed using all of the primer pairs as per the standard protocol required for Nanjing Kownsi’s high-fidelity PCR amplification kit. PCR system 20 μL, 2× Phanta ^®^ Max Master Mix 10 μL, upstream and downstream primers (10 μmol/L) 1 μL, template DNA (50 ng/μL) about 1 μL and ddH_2_O about 7 μL. PCR procedure: 95 °C for 5 min; then 35 cycles included reaction at 95 °C for 15 s, reaction at 51 °C–61 °C for 15 s (depending on the Tm value of each primer), and reaction at 72 °C for 30–60 s/kb (depending on the length of target sequence); last extension at 72 °C for 10 min; heat preservation at 12 °C. Detection of PCR products by 1–3% agarose gel electrophoresis [25].

The possible presence of nine R genes (*Xa1*, *Xa2*, *Xa3/Xa26*, *Xa4*, *Xa14*, *Xa23*, *Xa27*, *Xa31*(*t*) and *Xa45*(*t*)_4_) in HY-8 and its descendants was confirmed by comparing the amplicons in corresponding resistant and susceptible controls. Then we selected HY-8, F_2-3_, F_2-4_, No.10 and FD-3 as representative material. The genomic region of these five selected genotypes and the *O. officinalis* wall., (as control) were subjected to further amplification. The PCR product was collected and inserted in the T vector, and submitted to Beijing Offilla Biotechnology Co., Ltd. Kunming Branch for sequencing.

### 2.5. Statistical Analysis

The primary data analysis of field data and the marker data was performed by Microsoft Office Excel. The nucleotide data and primer designing were performed by the available online tools of the NCBI data base. The sequencing data was compared by biology software DNAMAN [26].

## 3. Results

### 3.1. Reactions of BB Pathogenic Strains on Wild Parent and Its Hybrid

**Figure 2:**
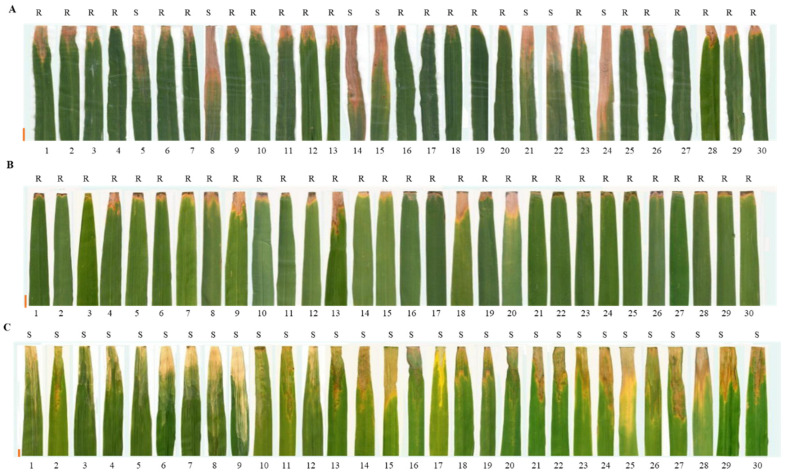
Identification and evaluation of disease assay for cultivated rice HY-8 (**A**), medicinal wild rice *Oryza officinalis* (**B**), and control variety 02428 (**C**) after inoculation with 30 *Xanthomonas* pathotypes, where, 1: C1, 2: C2, 3: C3, 4: C4, 5: C5, 6: C6, 7: C7, 8: C9, 9: Y8, 10: X1, 11: X6, 12: X9, 13: X10, 14: T7147, 15: PX099^A^, 16: PB, 17: Hz, 18: hzHJ19, 19: YM1, 20: YM187, 21: YJDP-2, 22: YJWS-2, 23: LN44, 24: HAN05-1, 25: HAN08-2, 26: HUB05-4, 27: YUN17-1, 28: YUN18-2, 29: YUN96-11, 30: YUN98-5, while S: susceptible; R: Resistant, side ruler indicates 1 cm of the lesion length.

The reactions of 30 bacterial pathogenic strains after 21 days of inoculation were studied to evaluate the resistance level of Mingding medicinal wild (MDMW) plant *O. officinalis* Wall., cultivated HY-8 parent and a susceptible control ‘02428’. The compatible reaction on the genotypes was observed with all 30 strains. The lesion length (LL) on the leaves of control plant ‘02428’ ranged from 15.4 cm to 25.4 cm. Hence, the MDMW was observed as resistant against all of the studied strains of BB pathogens (Table 2). The hybrid plant HY-8 was found to be resistant with an average 4.19 cm LL. Nonetheless, there was a strong reaction in HY-8 against seven pathovars including C5, C9, T7147, YJws-2, YJdp-2, PXO99^A^ and HAN05-1. The HAN05-1 with 15.97 cm LL showed the strongest reaction, followed by T7147 (10.3 cm) and C9 (10.1 cm) (Figure 2).

### 3.2. Reactions of BB Pathogens on 208 Progenies

The reaction response of all 208 hybrids and recombinants was evaluated against seven strong pathogenic strains. Based on their resistance response to seven pathogenic strains, all the genotypes were clustered into seven major groups which could be further divided into 21 subgroups (Table 3). Of the total 208 progenies, 61 were susceptible to all studied strains, while 37 showed resistance to one strain and were susceptible to others. There was no genotype resistant to HAN05-1 (Table 3). The representative plants of 21 resistance groups including individuals from F_1_, F_2_, BC_1_F_1_, BC_2_F_1_ and BC_3_F_1_ generations were further scored against the 30 pathogenic strains and the disease reaction as LL was measured (Supplementary Table S3). The 21 representative plants showed resistance against a minimum of 10 to a maximum of 29 pathogenic strains. There was no hybrid or recombinant individual that was resistant against the HAN05-1 isolate. The individuals in F_1_ and BC_1_F_1_ were resistant to most (more than 20) of the pathogenic strains (Supplementary Table S3). The F_1-1_, F_1-2-4_ and FC_7-11_ showed resistance to all pathogenic strains except HAN05-1. Among the two F_2_ plants, F_2-3_ showed resistance to 50% (15) of strains and susceptibility to the other 50% (15), on the other hand F_2-4_ was resistant to 27 strains (Supplementary Table S3).

### 3.3. Molecular Markers-Based Survey of BB Resistance Genes

**Figure 3:**
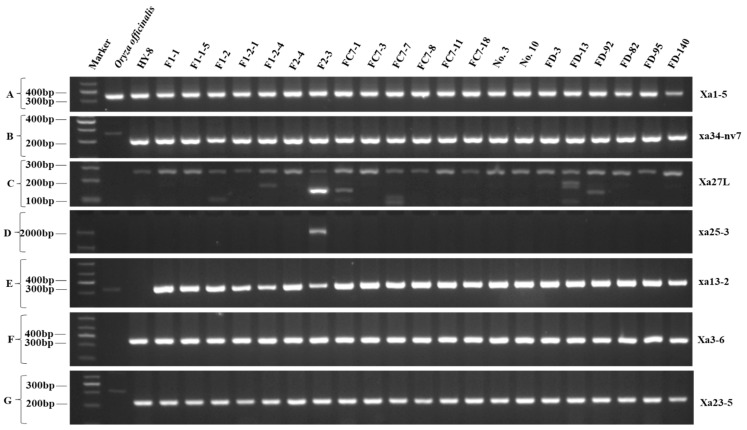
PCR amplicon based screening of genetic material, where (**A**–**E**) show five types of reactions, (**A**): reaction type 1 indicating the availability of ten fragments for primers *Xa1-3*, *Xa1-5*, *Xa3-1*, *Xa3-4*, *Xa3-5*, *Xa3-7*, *Xa3-8*, *Xa4-1*, *Xa4-6* and *Xa4-7,* (**B**) represents reaction type 2 indicating the availability of 14 fragments of primers *xa5L*, *Xa7-1*, *Xa7-2*, *Xa10-1*, *Xa10-2*, *Xa10-3*, *xa13-3*, *Xa21-2, xa25-1*, *xa34-nv7*, *Oso4g53050-1*, *RM27296*, *KGC3 16.3* and *Hxyj-1*, (**C**) represents the fragments for *Xa3-2*, *Xa4-4*, *xa13-4*, *xa25-3* and *Xa27L* primer pairs with inconsistent amplification, and (**D**) represents the only amplified fragment, (**E**) represents the fragments of *xa13-2* and *xa25-2* primers, (**F**) represents the non-amplified fragments of 24 primer pairs, while (**G**) represents the amplified fragments of remaining primers.

A molecular markers-based survey with 55 sequence tagged sites (STS) markers was performed for the parental, control and selected representative individuals of 21 resistant groups. The screening results of genotypes for the availability (+) or absence (−) of twenty R genes were evaluated (Table 4 and Figure 3). The gene of *Xa1*, *Xa4* and *X23* with ten primer pairs (Xa1-3, Xa1-5, Xa3-1, Xa3-4, Xa3-5, Xa3-7, Xa3-8, Xa4-1, Xa4-6 and Xa4-7) was detected in all individuals (Figure 3A). The genomic segments for *xa5*, *Xa7*, *Xa10*, *Xa27*, *Xa32*(*t*), *xa34*(*t*), *Xa38*(*t*), *xa42*(*t*) and *Xa45*(*t*)_11_ tested with fourteen primer pairs (xa5L, Xa7-1, Xa7-2, Xa10-1, Xa10-2, Xa10-3, xa13-3, Xa21-2, xa25-1, xa34-nv7, Oso4g53050-1, RM27296, KGC3 16.3, Hxyj-1) were not detected in any of individuals (Figure 3B) but may have their homologs. However, one or more segments of *Xa3*, *xa13* and *Xa25* were observed to be missing in recombinants.

The allele specific markers of resistant gene *Xa3* were tested. The *Xa3-2^d^* with 535 bp amplicon size was only identified in F_2-3_ and F_2-4_, and missing in all other individuals. Similarly, the 368 bp and 438 bp amplicons of *xa13*, and 954 bp amplicon of *Xa21* were missing in almost all individuals except *xa13-4* that was identified in F_2-3_ and F_2-4_ (Table 4, Figure 3C). Only a minor proportion of recombinants contained all of the five genomic segments of targeted genes (Figure 3B–E). The gene segment amplicons of xa13-2, xa25-2 were identified in *O. officinalis* but not in some of the offspring and the HY-8. The other 24 primer pairs which were not amplified in *O. officinalis* but found in progenies may indicate the absence of targeted segments in *O. officinalis* (Figure 3F,G). Nonetheless, the partial fragment or part of *Xa1*, *Xa3*, *Xa4*, *xa13* and *Xa25* reference genes were amplified in various recombinants. Hence, it could be concluded that F_2-3_ and F_2-4_ were commonly carrying nine R genes (*Xa1*, *Xa2*, *Xa3/Xa26*, *Xa4*, *Xa14*, *Xa23*, *Xa27*, *Xa31*(*t*), *Xa45*(*t*)*_4_*), while HY-8, No.10 and other decedents were carrying seven R genes (*Xa1*, *Xa2*, *Xa4*, *Xa14*, *Xa23, Xa31*(*t*) and *Xa45*(*t*)*_4_*) (Table 5).

### 3.4. Genomic Comparison of Polymorphic R Genes (Xa3/Xa26, Xa23, and Xa27)

Previous results showed that the HY-8, F_2-3_, F_2-4_, No.10 and the other progenies may contain nine R genes (*Xa1*, *Xa2*, *Xa3/Xa26*, *Xa4*, *Xa14*, *Xa23*, *Xa27*, *Xa31*(*t*) and *Xa45*(*t*)*_4_*) or their homologous gene. In order to further clarify these nine cases, which carry the genes, *O. officinalis*, HY-8, F_2-3_, F_2-4_, No. 10 and FD-3 representative genotypes were selected and their obtained PCR products were sequenced. The coding region of *Xa1*, *Xa2*, *Xa14*, *Xa31*(*t*) and the end of *Xa45*(*t*)*_4_* showed a number of deletions.

**Figure 4:**
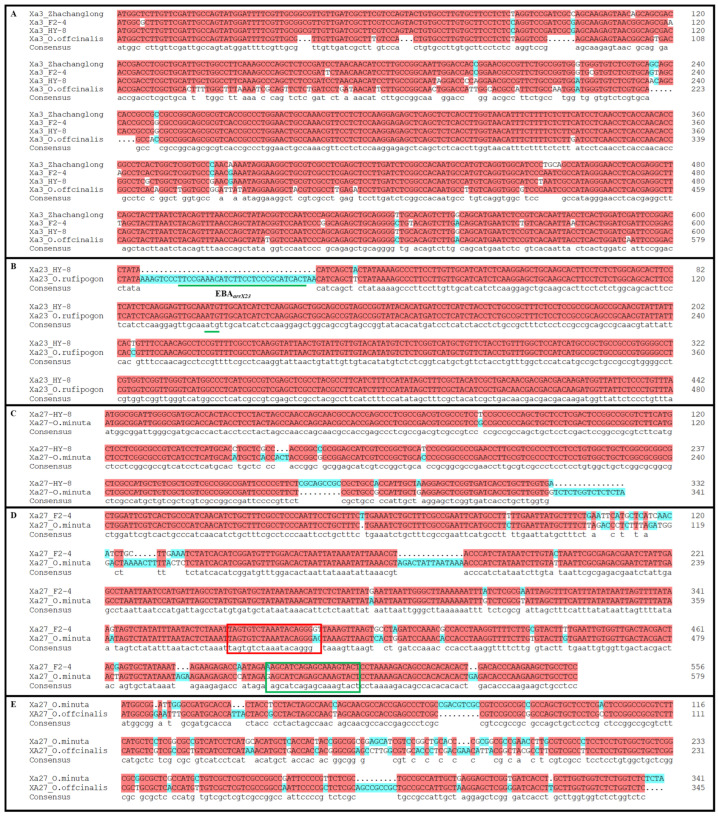
Comparative analysis of nucleotide sequences of *Xa3/Xa26*, *Xa23* and *Xa27*, where (**A**) indicates the comparison of genomic regions of *Xa3/Xa26* in different genotypes; (**B**) shows a comparison of the *Xa23* gene region between HY-8 and Zhachanglong; (**C**) indicates comparison results of coding region of *Xa27* gene in HY-8 and *O. minuta*; (**D**) indicates comparison of promoter regions of *Xa27* gene in F_2-4_ and *O. minuta*; (**E**) is the comparison of coding region of *Xa27* gene in *O. officinalis* and *O. minuta*, EBE*_avrXa23_* is a transcription activation effector that recognizes a specific DNA sequence on the promoter of *Xa23* gene, the red box indicates the *Xa27L* upstream primer binding region, and the green box shows *Xa27L* downstream primer binding region.

The *Xa1* had a deletion in 837 bp segment, *Xa2* had a deletion of 558 bp segment, *Xa14* type showed deletion of 405 bp, *Xa31*(*t*) showed deletion of 558 bp, and *Xa45*(*t*)*_4_* was missing 1116 bp. The genomic similarity between resistant donor wild parent and the HY-8 for *Xa1*, *Xa2*, *Xa14*, *Xa31*(*t*), and *Xa45*(*t*)*_4_* was up to 86.13%, 90.2%, 89.22%, 90.2% and 82.61%, respectively (Table 6, Figure 4). This may be an indication of the genes homology in HY-8.

The *Xa3/Xa26* region showed InDel and SNP mutations (Figure 3A) in the HY-8, F_2-3_, F_2-4_, No.10 and FD-3, and showed 96.05% similarity to the resistant donor. F_2-3_ and F_2-4_ seem to carry the two historical genomes AA and BB [22]. Wherein, AA was consistent with the HY-8, while for the BB genotype it showed 98.36% similarity to the resistant donor. Nonetheless, the downstream sequence of the *Xa3/Xa26* gene coding region (could be amplified by marker Xa3-KL2) could not be amplified in any medicinal rice plant. Hence, the primers near Xa3-KL3 within the coding region were designed (Figure 4A). The results showed that the target band could be amplified in *O. officinalis*, but the cloning and sequencing showed that the coding region sequence in *O. officinalis* was significantly different from that in F_2-3_ and F_2-4_, as well as that in the donor parent (Figure 4A), indicating that the BB genotype was not from two parents, but a new variation type. The *Xa4* sequence evaluation showed the 100% similarity of genomic and promoter regions among HY-8, F_2-3_, FD-3, F_2-4_, No.10 and the donor sequence in the resistant cultivar IR64, which indicated that the HY-8 and its hybrids contained *Xa4* gene. The sequence of *Xa23* in HY-8, F_2-3_, F_2-4_, No.10 and FD-3 showed 99.71% consistency to the donor wild rice varieties. In all five genotypes the DNA sequence has one SNP as a point mutation at 104 bp position (Figure 4B) i.e., nonsense mutation.

The *Xa27* sequence also showed the InDel and SNP mutation in HY-8, No.10 and FD-3 (Figure 4C), while having 89.74% consistency to the *O. minuta*. Its genomic region also supposed to be evolved from two parts from A and B genomes as in *Xa3/Xa26*. The genomic region in HY-8 was exactly the same as the *O. minuta* but the promoter region showed an inconsistent resistant due to a large difference among genotypes (Figure 4D). Further estimation from *O. officinalis* genome, as in Figure 3D showed a substantial matching of genome sequence in promoter region of *Xa27*, which could amplify the genomic region in F_2-3_ and F_2-4_ but could not amplify in the *O. officinalis*. The full-length amplification by primers Xa27-KL1 also could not amplify in wild rice. The coding region sequence of *O. officinalis* in F_2-3_ and F_2-4_ genotypes showed a larger variation and revealed only 78.27% similarity (Figure 4E). Hence, it could be concluded that the *O. officinalis*, HY-8, and the progenies contain only the homologous gene *Xa27*, while the F_2_ generation materials F_2-3_ and F_2-4_ may also contain *Xa27* resistance gene or susceptible allele *xa27*.

It can be seen from the above results that HY-8 and its hybrids contained the Xa4 gene. In addition, only the homologous genes of *Xa1*, *Xa2*, *Xa3/Xa26*, *Xa14*, *Xa23*, *Xa31*(*t*) and *Xa45*(*t*)*_4_* were carried. A small number of offspring contained the disease resistance gene *Xa27* or its susceptible allele *xa27*.

## 4. Discussion

Rice is an important crop contributing to global food security and grows in almost all ecosystems [27]. Rice production is being affected by various biotic and abiotic stresses. Among these stresses, BB caused by *Xoo* results in a significant reduction in global rice yield. It particularly has devastating effects in Asian countries including China, Pakistan and India [28]. Genetic diversity is always required for any successful rice breeding program [28]. Historically, BB has occurred epidemically and is now found in almost all major rice growing areas of Asia [27]. This study aimed to reveal the novel source of BB resistance in rice. Hence, a medicinal rice plant (*O. officinalis*) was evaluated and found as resistant even in the absence of historically known resistance (R) genes for BB.

Germplasm screening may lead to the identification of both narrow and broad sense resistance to various types of bacterial blast including leaf and neck blast [29,30]. In various studies, a geographically diverse mixture of blast isolates has been used to identify the stable QTL/gene(s) [31]. To date, forty-seven genes have been identified that induce resistance against broad spectrum or race specific resistance to *Xoo* [27]. Evolving environmental conditions could cause the emergence of new pathogenic variants. Hence, a fresh effort to reveal new sources of resistance in wild material may be helpful to generate longer-term resistance to BB in cultivated species.

The current study was conducted to screen the medicinal wild rice plants *O. officinalis* and its hybrids to characterize against BB. The evaluation of medicinal wild parent plants and their F_1_, F_2_ and backcross individuals for BB resistance by traits, specific morphological and gene specific molecular markers revealed novel sources of BB resistance. It further highlighted the chromosomal substitutions in genomic and promoter regions of inbred and recombinants with a reference to their parental wild genotypes.

The molecular marker survey revealed that *O. officinalis* does not contain the evaluated 20 reference genes. The markers-based amplification of genomic segments indicated the absence of a few targeted R genes segments in hybrid plant HY-8 but they were available in a few of the other progenies. Twenty-four primer pairs which were not amplified in *O. officinalis* and detected in progenies indicate the absence of targeted segments in *O. officinalis* (Figure 3F,G). Hence, it may be an indication of an unknown source of resistance in *O. officinalis*. Similar results were observed in a few recent studies, in which they used a molecular survey to screen the potentially resistant landraces and the tested genes were not found [28]. The identification of new genes and manipulation of *O. officinalis* in rice breeding may increase BB resistance since most of the genes are losing their durability and effectiveness [32].

The sharing of common R-gene segments or the availability of homolog genomic regions indicated a common pedigree. The sources of two genomes may have common parents with HY-8, and the other genotype as *O. officinalis*. Nonetheless, the absence of the downstream sequences of *Xa3/Xa26* gene coding region in *O. officinalis* indicated the availability of alternate and unrevealed causes of BB resistance. The sequencing analysis revealed the genetic differences between F_2-3_ from *O. officinalis* and F_2-4_ (Figure 4A). Hence, it illustrated that the two parental genotypes had a new type of mutation. We also observed that *O. officinalis*, HY-8 and their descendants did not contain the *Xa3/Xa26* gene but may contain *Xa3/Xa26* homologous genes.

The sequence similarity of genomic and promoter regions for *Xa4* and *Xa23* genes among HY-8, F_2-3_, FD-3, F_2-4_, No. 10 and the donor sequence in the resistant cultivar IR64, indicated the availability of these genes in HY-8 and its hybrids. It was observed that the five studied materials had exactly the same protein as in *O. rufipogon*, but their promoter regions were missing the 38 bp region, which created a resemblance to the susceptible *xa23* gene and allelic differences in the *Xa23* functional region (EBE avrXa23) [2,33]. Hence the recombinant progeny HY-8 contains a susceptible *xa23* and not the disease-resistant gene *Xa23*. Similarly, the resemblance of the promoter region of *Xa27* sequence as the *O. minuta* and observed variation in protein coding regions resulted in inconsistent resistance in the progeny. A previous study has also been reported for the promoter region variation of the *Xa27* [2,34]. The similarity with two differences in genomic region sequence of *Xa27* in susceptible cultivar IR24 and small grain wild has been reported. The promoter region of the susceptible genotype had an insertion of 10 bp at about 1.4 kb upstream of the ATG and an insertion of 25 bp before the TA frame. It may not only cause the variation in the promoter region but also affect the gene function from resistant *Xa27* to susceptible *xa27*. Furthermore, the similarity in the promoter region of *Xa27* with *O. officinalis* in F_2-3_ and F_2-4_ and a variation in the coding region was observed. Hence, we propose that *O. officinalis*, HY-8 and the progeny only possessed the homolog of *Xa27*, while the F_2_ generation may also contain *Xa27* resistance or susceptible allele *xa27*.

The wild species are valuable sources of potential genes for tolerance or resistance to abiotic and biotic stresses and are helpful for revealing the gaps in genetic diversity [28,35]. In the case of bacterial blight, many important genes such as *xa5*, *xa13* and *Xa21* have been identified from cultivated rice and wild species [2,36]. Previously, a dominant gene *Xa21* was identified from a wild rice parent *O. longistaminanta*, showed resistance to all six races of BB in Philippines [27]. However, it was defeated and broken down in other Asian countries such as Nepal, Thailand and India [27]. Another similar study was conducted on wild rice *O. malampuzhaensis* and *O. rufipogon*. They reported the susceptibility of *O. malampuzhaensis* for all 20 tested *Xoo* strains in the absence of *Xa21* but *O. rufipogon* showed resistance without *Xa21*, which may due to the availability of a new major gene. *O. rufipogon* was also identified as source of resistance to BB in China [27,37] and found the major BB resistance gene *Xa23* [37]. Other similar studies have been reported for the presence of BB resistance in *O. minuta* Presl. [38] and *O. latifolia* [39].

The current study revealed that in addition to carrying the *Xa1*, *Xa2*, *Xa3/Xa26*, *Xa14*, *Xa23*, *Xa31*(*t*) and *Xa45*(*t*)_4_ homologous genes, the hybrid of *O. officinalis* also contained the parts from the *xa27* susceptible region and had lesser parts from resistant *Xa27*, which may have come from the medicinal wild parent and directly contributed to BB resistance. The genotypes without the R genes exhibited a resistant response to *Xoo* and may be a valuable genetic resource for rice breeding for BB resistance for higher yield. The present study provides a reference for investigating medicinal rice for R gene(s) identification against BB using a forward genomics tool. The gene(s) linked to the molecular markers used for R gene assays can be used as a tool to validate the bi-parental or diverse mapping population. A genome-wide association analysis of BB resistance will help in the identification of DNA-markers.

## Figures and Tables

**Table 1:** Primer pairs used to amplify the total gene length of targeted R genes.

Gene/Donor	Primer Name	Forward Primer (5′-3′)	Reverse Primer (5′-3′)	Tm (°C)	Expected Product Size (bp)
*Xa1*/Huangyu; *Xa2*/Rantai Emas 2; *Xa14*/TN1; *Xa31*(*t*)/Changlong; *Xa45*(*t*)*_4_*/IRGC 102463	Xa1a *	CCACACGCCCCACACGCACTG	CCATCTCTGCAGCCCTCCCATACA	61	1823
	Xa1b *	GCAGCCCTCTTGCACACGCCATTGG	CCGGTACATCAGTATTGTCCATCGG	55	552
	Xa1-1 *	AGGAACTGTGTATATCGTGC	TAGGATACGAGTTGGTGGAT	55	989
	Xa1-2 *	ACACCGAAACACTACAATCA	ACAGTAGGATACGAGTTGGT	55	444
	Xa1-3 *	ACCAACTCGTATCCTACTGT	TGGTATGATCGAACTGTCAC	55	191
	Xa1-4 *	CCAAACAGATACCAACTCCT	GTGGTATGATCGAACTGTCA	55	287
	Xa1-5 *	ATCCACCAACTCGTATCCT	TGTTGTTTACAGGAGAGCAA	55	356
	Xa1-KL1 *	TCAGACGATTAATCCACGACGA	TCTTTTCTGGGAGCTGTCTTGA	59	7400
*Xa3/Xa26*/Changlong	Xa3-1 *	ATGTGGCAGACTTTGGTATT	GCTCATATACCACGAGAGAG	55	605
	Xa3-2 *	CAGTGATTCATCGCTCTCTC	TAATGACGTGTGTGAGGTTT	55	535
	Xa3-3 *	CAATCGTTGCTGTTCTAACC	ATTAAGTAGCTGAAGCCTCG	55	853
	Xa3-4 *	AACGTTGGTAACAATAGCCT	CAGTGGAATCTGACCAAAGA	55	258
	Xa3-5 *	TCCTGCTACTGAAAGGAAAC	CCGTTAGTGAGTTCATGCTA	55	72
	Xa3-6 *	CTACACAATCGTTGCTGTTC	TTCACAGTGACGATCAAGTT	55	321
	Xa3-7 *	CCAGTATGGATTTTCGTTGC	TTAAGGTGTTGGAGGATTGG	55	692
	Xa3-8 *	CCTTGGGTGGGAATAACTTT	GGTAATGATCCATCCAGCAA	55	253
	Xa3-9 *	ACCTTAACTTTCAGGCCAAT	AAAGTTATTCCCACCCAAGG	55	317
	Xa3-KL2 *	ATCGTTGCTGTTCTAACCACC	TCGTCGTTTAGTGTCCACCTC	60	4207
	Xa3-KL3 *	TCACACACAACCAGACATGG	ATACCACGAGAGAGCGATGA	58	3454
*Xa4*/IR64	Xa4-1 *	TGGACATCATCGTTTTCACT	GTCAGCATATACGTTCCACT	55	623
	Xa4-2 *	ATGTTTCTTTGTATGCAGCG	AGCATATACGTTCCACTTCC	55	743
	Xa4-3 *	CAACGACGAGTGATTCTTTG	AGTACGGCCTGCTATATTTG	55	205
	Xa4-4 *	ACACATGCATGGTGGATATT	ATCCGAGACCTTTTATCTGC	55	703
	Xa4-5 *	ATACAGTACCGATACGAGGT	TATATGTACGTTTGCTGCGA	55	151
	Xa4-6 *	GAATGAGAGTCAGAAGGGAC	CGCTGCATACAAAGAAACAT	55	187
	Xa4-7 *	TTACTTGTTACGGTGGTAGC	GGTTTACCTATTGCATTGGC	55	157
	Xa4-8 *	TAGTCGTCATACAGTACCGA	GTATATGTACGTTTGCTGCG	55	160
	Xa4-9 *	TGACATGAACTACATCGACC	CATCCTCATAATGGCTGACA	55	164
	Xa4-KL3 *	ACTTCTAATATGGTAGTCGTCA	AGGTAGATTTGCACCTCTGAT	50	5701
*xa5*/IRBB5	xa5L	CCGGAGCTCGCCATTCAAGTTCTTG	TGCTCTTGACTTGGTTCTCC	55	145
*Xa7*/IRBB7	Xa7-1 *	CATCCTGATCGTATGCCCGT	GCGACGAGGGCAATAGACAT	58	248
	Xa7-2 *	GACTGCTGACCGTCAACTCC	GCCACCGATGAGGTAATCCTG	58	242
*Xa10*/IRBB10	Xa10-1 *	ATCGGGTTCCTCTACATCTC	AGCTATACGGGCATAAGAAG	55	148
	Xa10-2 *	TGTCGCAATCACTTCAATTAC	GAGAGGTAGAAGAGTATGGC	55	477
	Xa10-3 *	CTCCTTCTTATGCCCGTATAG	CGCCGGTTTCTCTTTATTAAC	55	415
*xa13*/BJ1	xa13-1 *	GCTTAGTCACTTGATTGCAC	CCTCTCTCCACTACTCTGAA	55	309
	xa13-2 *	AAAACATCTTGGCATGTTGG	GTGCAATCAAGTGACTAAGC	55	331
	xa13-3 *	GCTTTAGGATTAGCGGGTTA	TGGAATGCTGATCAATGGAA	55	368
	xa13-4 *	AGCACTTAAGCCTTTCTCTC	CTAGAAGCATCAAAAGCGTG	55	438
*Xa21/O. longistaminata*	Xa21-1 *	GAAGCACTACGAAATATGCG	ATTGCAGTGTAGAGCAGAAA	55	675
	Xa21-2 *	CAGCAAGTCCTTCCAGTATT	AATCGGGTCTGAATGTACTG	53	954
	Xa21-3 *	AAAAGCAACAGATGGTTTCG	ATCAATGAGGTCCCATCAAC	55	516
	Xa21-4 *	GGAGGGATCAATACCACAAG	CTGCTAAGGATGTGGGTATC	55	245
	Xa21-5 *	CCTCGATGTTGTCCATTACT	AGCTTTAGTACCTTCACTGC	55	305
	Xa21-6 *	CAGTACATTCAGACCCGATT	ATCCGGAGAGATTCTGTTTG	55	269
	Xa21-KL4 *	TTGACGAAGACGACCGCTAC	TGCGGTGTGGCAATTCAGAG	58	4583
*Xa23/O. rufipogon*	Xa23-1 *	CCGGTATACACATGATCCTC	CAGTTAATACCTTGAGGCGA	55	111
	Xa23-2 *	TAGCTTGTGTTGTGAGTTGT	TGGAATCCCAGAATTCGATG	55	606
	Xa23-3 *	CGCCTCAAGGTATTAACTGT	AATAACCATCTTGTCGTCGT	55	204
	Xa23-4 *	ACGACGACAAGATGGTTATT	GTGACTGATCACTACACACA	55	337
	Xa23-5 *	AAACAACCATTACAGAGCCA	AGGAGGAGGTAAGGGATAGA	55	222
	Xa23-KL13 *	AATTATGCGGCATCACTAACA	TGGATGAGGATATGATGAGC	55	797
*Xa25*/Minghui 63	xa25-1 *	TGTGTGAGAGAAGTTCCAAG	GAGCAGTTTGTGATTTGAAGA	51	2187
	xa25-2 *	GTGTGTGACCACATGAATTG	TGAATACAACAGAAGCGGAA	51	847
	xa25-3 *	ACCACAACTAAGACATTCCC	TGAGCAGTTTGTGATTTGAAG	52	1996
*Xa27/O. minuta*	Xa27L	TAGTGTCTAAATACAGGGACT	GAGTACTTTGCTCTGATGCTC	56	149
	Xa27-KL1 *	CTGGATTCGTCACTGCCCAT	AAAATCGGCCCAAACAACGG	60	1148
	Xa27-KL7 *	ATGGCGGATTGGGCGATG	GAGACCAGAGACCACCAAGC	60	337
*Xa32*(*t*)*/O. australiensis*	RM27296	GGGTCTTTGTACACATTCTTGTGG	CTTGAAGGATGAGCAGTATCTCG	55	500
*xa34*(*t*)/BG1222	xa34-nv7	GTCTTGGGTGGAAGTCTGACCTC	GGGTAGGTCTGTTTGCAAGAGTTG	55	411
*Xa38*(*t*)*/O. australiensis*	Oso4g53050-1	TCTTCTATTGCTAACATTGGTG	TCGCATTCATTTTCAGAG	55	269
*xa42*(*t*)/Baixiangzhan	KGC3_16.3	ATTAGAGTATCCACCAATAAGCCCG	GAGGTAAGATGAGATCGTGTAGGAG	55	247
*Xa45*(*t*)*_11_/O. rufipogon* from Yuanjiang	Hxjy-1	GTCTTGGGTGGAAGTCTGACCTC	GGGTAGGTCTGTTTGCAAGAGTTG	55	169

*: Represents primers designed according to gene nucleotide sequences.

**Table 2:** Average lesion length (in cm) and standard deviation in resistant and susceptible parents and control against various pathogenic strains evaluated against 30 pathogenic strains.

Strain	Susceptible Control	*O. officinalis*	HY-8
	Mean ± SD	Mean ± SD	Mean ± SD
C1	18.50 ± 1.26	0.90 ± 0.36	6.00 ± 0.41
C2	16.43 ± 0.65	1.20 ± 0.16	5.27 ± 0.71
C3	16.8 ± 1.39	0.37 ± 0.09	3.77 ± 0.38
C4	17.13 ± 1.25	2.47 ± 1.01	0.80 ± 0.22
C5	21.00 ± 0.83	1.07 ± 0.39	8.17 ± 0.59
C6	16.40± 1.67	0.60 ± 0.08	2.43 ± 1.37
C7	23.67 ± 3.18	1.30 ± 0.73	5.73 ± 0.56
C9	25.40 ± 2.29	1.50 ± 0.64	10.10 ± 0.62
Y8	20.57 ± 2.03	3.33 ± 0.54	1.57 ± 0.33
X1	18.03 ± 1.09	1.50 ± 0.36	2.33 ± 0.71
X6	15.90 ± 1.39	0.83 ± 0.33	2.57 ± 0.83
X9	18.03 ± 0.95	1.47 ± 0.34	2.40 ± 0.29
X10	17.53 ± 1.6	3.73 ± 1.11	4.53 ± 1.58
T7147	21.73 ± 1.39	0.63 ± 0.21	10.30 ± 2.97
PXO99^A^	19.03 ± 2.83	0.73 ± 0.24	9.17 ± 1.57
PB	17.63 ± 0.54	0.77 ± 0.12	0.70 ± 0.41
HZ	18.46 ± 1.25	1.03 ± 0.4	0.83 ± 0.29
Hzhj19	17.90 ± 0.82	2.70 ± 1.42	2.37 ± 0.82
Ym1	15.90 ± 1.1	1.07 ± 0.17	1.00 ± 0.62
Ym187	16.83 ± 1.73	2.90 ± 0.86	0.60 ± 0.71
YJdp-2	16.73 ± 0.41	0.57 ± 0.33	8.50 ± 0.41
YJws-2	21.33 ± 1.07	1.13 ± 0.34	7.23 ± 0.53
LN44	16.53 ± 0.69	0.80 ± 0.08	3.67 ± 0.85
HAN05-1	18.50 ± 0.78	1.27 ± 0.21	15.97 ± 4.12
HAN08-2	15.40 ± 0.99	0.57 ± 0.29	0.83 ± 0.41
HUB05-4	17.60 ± 0.96	0.63 ± 0.33	3.87 ± 0.53
YuN17-1	18.83 ± 0.54	0.47 ± 0.33	0.27 ± 0.12
YuN18-2	16.20 ± 1.04	0.63 ± 0.05	2.73 ± 2.31
YuN96-11	15.77 ± 2.99	1.17 ± 0.77	1.10 ± 0.65
YuN98-5	17.20 ± 1.85	0.80 ± 0.45	1.03 ± 0.5

**Table 3:** Disease reaction study for seven strong pathogenic strains on *O. officinalis*, its hybrids and recombinants.

	Sample	T7147	C5	C9	YJws-2	YJdp-2	PXO99^A^	HAN05-1
Resistant Group 1	1	R	R	R	R	R	R	S
	2	R	R	R	R	R	R	S
	7	R	R	R	R	R	R	S
	15	R	R	R	R	R	R	S
	17	R	R	R	R	R	R	S
	19	R	R	R	R	R	R	S
	F1-1	R	R	R	R	R	R	S
	F1-1-2	R	R	R	R	R	R	S
	F1-1-3	R	R	R	R	R	R	S
	F1-1-4	R	R	R	R	R	R	S
	F1-1-6	R	R	R	R	R	R	S
	F1-1-7	R	R	R	R	R	R	S
	F1-2-2	R	R	R	R	R	R	S
	F1-2-3	R	R	R	R	R	R	S
	F1-2-4	R	R	R	R	R	R	S
	F1-2-5	R	R	R	R	R	R	S
	F1-2-6	R	R	R	R	R	R	S
	F2-2	R	R	R	R	R	R	S
	FC1-1-7	R	R	R	R	R	R	S
	FC7-1	R	R	R	R	R	R	S
	FC7-2	R	R	R	R	R	R	S
	FC7-4	R	R	R	R	R	R	S
	FC7-5	R	R	R	R	R	R	S
	FC7-10	R	R	R	R	R	R	S
	FC7-11	R	R	R	R	R	R	S
	FC7-16	R	R	R	R	R	R	S
	FC7-18	R	R	R	i	R	R	S
Resistant Group 2	3	R	R	R	R	R	S	S
	4	R	R	R	R	R	S	S
	6	R	R	R	R	R	S	S
	11	R	R	R	R	R	S	S
	FC7-8	R	R	R	R	R	S	S
	FC7-9	R	R	R	R	R	S	S
	FC7-12	R	R	R	R	R	S	S
	FC7-15	R	R	R	R	R	S	S
	FC7-19	R	R	R	R	R	S	S
	FC7-23	R	R	R	R	R	S	S
	FC7-24	R	R	R	R	R	S	S
	FC7-25	R	R	R	R	R	S	S
	FC7-26	R	R	R	R	R	S	S
Resistant Group 3	5	R	R	S	R	R	R	S
	9	R	R	S	R	R	R	S
	13	R	R	S	R	R	R	S
	FC7-13	R	R	S	R	R	R	S
	FC7-14	R	R	S	R	R	R	S
	FC7-21	R	R	S	R	R	R	S
	FC7-20	R	R	S	R	R	R	S
	FC11	R	R	S	R	R	R	S
	FC7-22	R	R	S	R	R	R	S
Resistant Group 4	F1-2-1	R	R	R	R	S	S	S
	FC7-7	R	R	R	R	S	S	S
	FD-2	R	R	R	R	S	S	S
	FD-26	R	R	R	R	S	S	S
	FD-29	R	R	R	R	S	S	S
	FD-50	R	R	R	R	S	S	S
	FD-106	R	R	R	R	S	S	S
	FD-107	R	R	R	R	S	S	S
Resistant Group 5	FC7-3	S	S	R	R	R	R	S
	FD-1	S	S	R	R	R	R	S
	FD-86	S	S	R	R	R	R	S
	FD-96	S	S	R	R	R	R	S
Resistant Group 6	F_2-4_	R	R	R	S	S	R	S
	FD-25	R	R	R	S	S	R	S
	FD-28	R	R	R	S	S	R	S
	FD-30	R	R	R	S	S	R	S
	FD-51	R	R	R	S	S	R	S
	FD-69	R	R	R	S	S	R	S
	FD-72	R	R	R	S	S	R	S
	FD-105	R	R	R	S	S	R	S

**Table 4:** The molecular markers amplification in *O. officinalis* and its offspring.

Gene	Primer Name	Expected Product Size (bp)	*O. officinalis*	HY-8	F_2-3_	F_2-4_	No. 10	Other Progenies
*Xa1*	Xa1a	1823	-	+	+	+	+	+
	Xa1b	552	-	+	+	+	+	+
	Xa1-1	989	-	+	+	+	+	+
	Xa1-2	444	-	+	+	+	+	+
	Xa1-3	191	+	+	+	+	+	+
	Xa1-4	287	-	+	+	+	+	+
	Xa1-5	356	+	+	+	+	+	+
*Xa3*	Xa3-1	605	+	+	+	+	+	+
	Xa3-2	535	-	-	+	+	-	-
	Xa3-3	853	-	+	+	+	+	+
	Xa3-4	258	+	+	+	+	+	+
	Xa3-5	72	+	+	+	+	+	+
	Xa3-6	321	-	+	+	+	+	+
	Xa3-7	692	+	+	+	+	+	+
	Xa3-8	253	+	+	+	+	+	+
	Xa3-9	317	-	+	+	+	+	+
*Xa4*	Xa4-1	623	+	+	+	+	+	+
	Xa4-2	743	-	+	+	+	+	+
	Xa4-3	205	-	+	+	+	+	+
	Xa4-4	703	-	+	+	+	+	+
	Xa4-5	151	-	+	+	+	+	+
	Xa4-6	187	+	+	+	+	+	+
	Xa4-7	157	+	+	+	+	+	+
	Xa4-8	160	-	+	+	+	+	+
	Xa4-9	164	-	+	+	+	+	+
*xa5*	xa5L	145	-	-	-	-	-	-
*Xa7*	Xa7-1	248	-	-	-	-	-	-
	Xa7-2	242	-	-	-	-	-	-
*Xa10*	Xa10-1	148	-	-	-	-	-	-
	Xa10-2	477	-	-	-	-	-	-
	Xa10-3	415	-	-	-	-	-	-
*xa13*	xa13-1	309	-	+	+	+	+	+
	xa13-2	331	+	-	+	+	+	+
	xa13-3	368	-	-	-	-	-	-
	xa13-4	438	-	-	+	+	-	-
*Xa21*	Xa21-1	675	-	+	+	+	+	+
	Xa21-2	954	-	-	-	-	-	-
	Xa21-3	516	-	+	+	+	+	+
	Xa21-4	245	-	+	+	+	+	+
	Xa21-5	305	-	+	+	+	+	+
	Xa21-6	269	-	+	+	+	+	+
*Xa23*	Xa23-1	111	-	+	+	+	+	+
	Xa23-2	606	-	+	+	+	+	+
	Xa23-3	204	-	+	+	+	+	+
	Xa23-4	337	-	+	+	+	+	+
	Xa23-5	222	-	+	+	+	+	+
*Xa25*	xa25-1	2187	-	-	-	-	-	-
	xa25-2	847	+	-	-	+	-	+
	xa25-3	1996	-	-	+	-	-	-
*Xa27*	Xa27L	149	-	-	+	+	-	-
*Xa32*(*t*)	RM27296	500	-	-	-	-	-	-
*xa34*(*t*)	xa34-nv7	411	-	-	-	-	-	-
*Xa38*(*t*)	Oso4g53050-1	269	-	-	-	-	-	-
*xa42*(*t*)	KGC3_16.3	247	-	-	-	-	-	-
*Xa45*(*t*)_11_	Hxjy-1	169	-	-	-	-	-	-

**Table 5:** Genes list predicted availability in *O. officinalis*, its hybrids and recombinant.

Description of Sample	Resistance Gene
*O. officinalis*	None of the tested genes
HY-8	*Xa1*, *Xa2*, *Xa4*, *Xa14*, *Xa23*, *Xa31*(*t*), *Xa45*(*t*)*_4_*
F_2-3_	*Xa1*, *Xa2*, *Xa3/Xa26*, *Xa4*, *Xa14*, *Xa23*, *Xa27*, *Xa31*(*t*), *Xa45*(*t*)_4_
F_2-4_	*Xa1*, *Xa2*, *Xa3/Xa26*, *Xa4*, *Xa14*, *Xa23*, *Xa27*, *Xa31*(*t*), *Xa45*(*t*)_4_
No. 10	*Xa1*, *Xa2*, *Xa4*, *Xa14*, *Xa23*, *Xa31*(*t*), *Xa45*(*t*)*_4_*
Other descendants	*Xa1*, *Xa2*, *Xa4*, *Xa14*, *Xa23*, *Xa31*(*t*), *Xa45*(*t*)*_4_*

**Table 6:** Consistency of gene coding regions among *O. officinalis*, HY-8 and its hybrid.

Gene Name	*O. officinalis* Wall	HY-8	F_2-3_	F_2-4_	No. 10	FD-3
*Xa1*	/	86.13%	86.13%	86.13%	86.13%	86.13%
*Xa2*	/	90.20%	90.20%	90.20%	90.20%	90.20%
*Xa3/Xa26*	94.50%	96.05%	96.05%/98.36%	96.05%/98.36%	96.05%	96.05%
*Xa4*	/	100%	100%	100%	100%	100%
*Xa14*	/	89.22%	89.22%	89.22%	89.22%	89.22%
*Xa23*	/	99.71%	99.71%	99.71%	99.71%	99.71%
*Xa27*	78.27%	89.74%	89.74%/100%	89.74%/100%	89.74%	89.74%
*Xa31*(*t*)	/	90.20%	90.20%	90.20%	90.20%	90.20%
*Xa45*(*t*)*_4_*	/	82.61%	82.61%	82.61%	82.61%	82.61%

/: Represents the absence of a target gene coding region.

## Data Availability

Not applicable.

## Supplementary Materials

The supporting information can be downloaded at: https://www.mdpi.com/article/10.3390/life12060827/s1.
